# Human Hair Follicle-Derived Mesenchymal Stromal Cells from the Lower Dermal Sheath as a Competitive Alternative for Immunomodulation

**DOI:** 10.3390/biomedicines10020253

**Published:** 2022-01-24

**Authors:** Beatriz Hernaez-Estrada, Ainhoa Gonzalez-Pujana, Andoni Cuevas, Ander Izeta, Kara L. Spiller, Manoli Igartua, Edorta Santos-Vizcaino, Rosa Maria Hernandez

**Affiliations:** 1 School of Biomedical Engineering, Science and Health Systems, Drexel University, Philadelphia, PA 19104, USA; bh663@drexel.edu (B.H.-E.); kls35@drexel.edu (K.L.S.); 2 NanoBioCel Research Group, Laboratory of Pharmaceutics, School of Pharmacy, University of the Basque Country (UPV/EHU), Paseo de la Universidad 7, 01006 Vitoria-Gasteiz, Spain; ainhoa.gonzalez@ehu.eus (A.G.-P.); manoli.igartua@ehu.eus (M.I.); 3 Biomedical Research Networking Centre in Bioengineering, Biomaterials and Nanomedicine (CIBER-BBN), Institute of Health Carlos III, 28029 Madrid, Spain; 4 Bioaraba, NanoBioCel Research Group, 01006 Vitoria-Gasteiz, Spain; 5 Clínica Ercilla, 48009 Bilbao, Spain; laboratorio@clinicaercilla.com; 6 Tissue Engineering Group, Biodonostia Health Research Institute, 20014 Donostia-San Sebastián, Spain; ander.izeta@biodonostia.org; 7 Department of Biomedical Engineering and Sciences, School of Engineering, Tecnun-University of Navarra, 20009 Donostia-San Sebastián, Spain

**Keywords:** immunomodulation, hair follicle, MSCs, macrophages, regulatory T cells, PBMC, immunoevasive

## Abstract

Mesenchymal stromal cells (MSCs) have unique immunomodulatory capacities. We investigated hair follicle-derived MSCs (HF-MSCs) from the dermal sheath, which are advantageous as an alternative source because of their relatively painless and minimally risky extraction procedure. These cells expressed neural markers upon isolation and maintained stemness for a minimum of 10 passages. Furthermore, HF-MSCs showed responsiveness to pro-inflammatory environments by expressing type-II major histocompatibility complex antigens (MHC)-II to a lesser extent than adipose tissue-derived MSCs (AT-MSCs). HF-MSCs effectively inhibited the proliferation of peripheral blood mononuclear cells equivalently to AT-MSCs. Additionally, HF-MSCs promoted the induction of CD4+CD25+FOXP3+ regulatory T cells to the same extent as AT-MSCs. Finally, HF-MSCs, more so than AT-MSCs, skewed M0 and M1 macrophages towards M2 phenotypes, with upregulation of typical M2 markers CD163 and CD206 and downregulation of M1 markers such as CD64, CD86, and MHC-II. Thus, we conclude that HF-MSCs are a promising source for immunomodulation.

## 1. Introduction

Mesenchymal stromal cells (MSCs) are proliferative and multipotent cells that exist in most tissues of the body and show numerous therapeutic properties. As their immunomodulatory potential has become increasingly appreciated through hundreds of clinical trials—reviewed in [1]—in recent years, a number of MSC cell therapies have been approved for clinical use, including Temcell^®^, Alofisel^®^, and Remestemcel-L—reviewed in [1,2].

MSCs have two important characteristics that make them an attractive therapeutic option for a wide range of inflammatory and immune-mediated diseases: immune evasion and immunomodulation. Their ability to evade immune rejection results from relatively low levels of major histocompatibility class I (MHC-I) expression and absence of the type-II (MHC-II) [3]. The immunomodulatory abilities of MSCs are numerous in that they regulate both adaptative and innate responses, relaying on paracrine factors and cellular contact for their function. MSCs inhibit the proliferation of lymphocytes, induce the generation of regulatory T cells, and skew macrophages towards a more pro-regenerative and pro-tolerogenic phenotype [3,4,5]. In humans, one of the main effectors in MSC-mediated immunomodulation is indoleamine 2,3-dioxygenase (IDO), a metabolic enzyme that is secreted by MSCs upon exposure to inflammatory conditions [6]. However, the clinical potential of MSCs remains limited by numerous obstacles, including (i) the invasive and painful harvesting procedure with possible complications; (ii) their gradual loss of “stemness” and immunomodulatory properties during in vitro expansion; and (iii) their loss of immune evasiveness in inflammatory environments [7].

MSCs can be harvested from almost every tissue using their plastic-adherence properties. Historically, bone marrow was the first source from which MSCs were obtained [8]. MSCs have since been isolated from other tissue sources, reviewed in [9]. Recently, adipose tissue-derived MSCs (AT-MSCs) have been utilized to almost the same extent as bone marrow-derived MSCs (BM-MSCs) because they are easier to isolate in higher numbers [10]. However, the procedures to harvest these cells are still relatively harmful, painful, and invasive for the patient. Therefore, there is a need to evaluate the immunomodulatory potential of MSCs derived from other, more easily accessible sources.

The hair follicle is one of the main appendages of the skin and the source of two different types of multipotent cells: epidermal-origin stem cells and dermal-origin MSCs—HF-MSCs from now on—reviewed in [11,12]. HF-MSCs can be obtained from the dermal papilla region and from the dermal sheath [13]. One of the main characteristics of the HF-MSCs that differs from MSCs of other sources is their possible neural crest origin [14,15]. Consequently, this difference of origin could lead to the development of novel characteristics [16]. This could be attributed to the possible presence of neural markers such as SOX-2, CD271, and CD56 [15,17]. To date, only very few studies have described the potential of HF-MSCs [18,19,20,21,22]. Recently, Li et al. isolated MSCs from the outer root sheath of the epilated hair follicle and showed that these cells might display immunomodulatory potential in that they reduced secretion of pro-inflammatory cytokines in cultures of pro-inflammatory macrophages and increased secretion of IL10 and expression of the M2 macrophage marker CD163 [23]. However, the immunomodulatory potential of HF-MSCs has not yet been explored in detail, and whether they display the same capabilities of other sources of MSCs is not known.

In the present study, we sought to explore the immunomodulatory properties of primary HF-MSCs obtained from human donors undergoing hair transplant therapy. We first characterized their location within the hair follicle, their expression of neural markers, and their stemness at both early and late passages in vitro. Subsequently, we evaluated the immunomodulatory capacity of HF-MSCs in comparison to AT-MSCs in terms of their ability to suppress proliferation of peripheral blood mononuclear cells (PBMCs), to stimulate induction of regulatory T cells, and to promote M2 polarization of macrophages. The results comprehensively demonstrated that HF-MSCs represent an effective and accessible source of MSCs with immunomodulatory potential comparable to that of AT-MSCs.

## 2. Materials and Methods

### 2.1. Experimental Procedures

#### 2.1.1. HF-MSCs

Hair follicles were obtained from occipital scalps of routine hair transplant procedures with the application of FUE technique (Clínica Dermatológica Ercilla, Spain) following informed consent under an approved protocol (M30_2019_054, M10_2019_053). Each collection consisted of five hair follicles per patient. This procedure, which is minimally invasive, was performed under local anesthesia.

To obtain the HF-MSCs, the explant attachment method was used. Here, we made sure only the lower part of the hair follicle was attached to the plate. Hair follicles were incubated at 37 °C in a humidified atmosphere containing 5% CO_2_ with Dulbecco’s modified Eagle’s medium (DMEM) (Life Technologies, Bleiswijk, The Netherlands) supplemented with 30% human serum and 10% antibiotic/antimycotic solution (Life Technologies, Bleiswijk, The Netherlands). We also took advantage of the fact that MSCs are able to attach to the plastic to remove all the cells that were not attaching by changing the media every 3–4 days. Around the 15th day, HF-MSCs were released from the hair follicles. These cells were considered to be in passage 0. Subsequently, the cells were harvested using trypsin (Life Technologies, Bleiswijk, The Netherlands) and expanded. To maintain consistency and for a thorough comparison, pools of no more than 5 different patients’ cells were used. All subsequent characterization experiments were performed using both passages 2 and 10.

#### 2.1.2. PBMCs, T-Lymphocytes, and Monocytes

PBMCs were isolated from the blood of healthy donors (San Jose Medical Center, Vitoria, Spain), following informed consent under a protocol approved by the research ethics committee of the Basque Country and that of Txagorritxu University hospital (CES-BIOEF 2017-26, CEIC Áraba Expte 2017-025). PBMCs were separated from the whole blood by a density gradient centrifugation. Then CD4+ T cells and CD14+ monocytes were purified from PBMCs using CD4 and CD14 MicroBeads respectively (Mitenyi Biotec, Bergisch Gladbach, Germany).

### 2.2. Expansion and Preparation of MSCs

AT-MSC were purchased from ATCC (Manassas, VA, USA) and were cultured with the recommended medium. HF-MSCs were grown in DMEM supplemented with 10% of Fetal Bovine Serum (FBS) (Life Technologies, Bleiswijk, The Netherlands) and 1% antibiotic/antimycotic solution. To generate IFNγ-licensed MSCs, cells were treated with IFNγ (100 ng/mL) (Sigma-Aldrich, Saint Louis, MO, USA) in complete media for 72 h.

### 2.3. HF-MSCs Characterization

#### 2.3.1. Phenotype Analysis

For the analysis of neural markers, hair follicles were incubated in a 0.2 mg/mL collagenase solution (Sigma-Aldrich, Madrid, Spain). Then, hair follicles were homogenized using a gentleMACS^TM^ tissue dissociator (Miltenyi Biotec, Bergisch Gladbach, Germany). Cells were stained with anti-CD271-APC, anti-CD56-PE, and anti-CD90-PE-VIO770 or with the corresponding isotype controls following manufacturer instructions (Miltenyi Biotec, Bergisch Gladbach, Germany). Subsequently, the cells were fixed and permeabilized using an Intracellular Staining Buffer Set and cells were then stained with anti-SOX2-FITC or with the corresponding isotype control following manufacturer instructions (Miltenyi Biotec, Bergisch Gladbach, Germany) and analyzed by flow cytometry. To determine if the cells were able to maintain stemness even in late passages, cells in passage 2 and 10 were stained with the MSC Phenotyping kit, anti-MHC-I-PeVio770, and anti-MHC-II-VioBlue or corresponding isotype controls following the manufacturer’s instructions (Miltenyi Biotec, Bergisch Gladbach, Germany). Data were acquired by flow cytometry using the MACSQuant^®^ analyzer (Miltenyi Biotec, Bergisch Gladbach, Germany). Data were analyzed using MACSQuantify software (Miltenyi Biotec, Bergisch Gladbach, Germany) and FlowJo^TM^ v10.6.2 Software (BD Life Sciences, Ashland, OR, USA).

CD105 and CD73 expression were only measured in HF-MSCs after cultured until passage 2 and passage 10 to certify that the cells could be considered MSCs. As in all of the cells the three markers—CD90, CD105, and CD73—were co-expressed, we picked just one to conduct the characterization analysis in freshly harvested HF-MSCs. For that, cells were stained with CD90, CD105, and CD73 with the same staining protocol we were using for the immunohistochemistry—described in 2.3.5. The cells stained with CD90 showed a brighter signal than the cells stained with CD105 and CD73. For that reason, CD90 marker was chosen to perform the immunohistochemistry assay. To maintain the same marker through the study, CD90 was also used to gate freshly harvested HF-MSCs in the neural characterization assay.

#### 2.3.2. Differentiation Capacity of MSCs

For osteogenic and adipogenic differentiation, HF-MSCs were seeded at a density of 2 × 10^6^ cells/well and cultured in differentiation medium for 3 weeks. Osteogenic differentiation medium: basal medium was supplemented 0.05 mM with L-ascorbic acid (Merck Life Science, Madrid, Spain), 20 nM β-glycerophosphate (Merck Life Science, Madrid, Spain) and 100 nM dexamethasone (Merck Life Science, Madrid, Spain). Adipogenic differentiation medium: basal medium was supplemented with 0.5 µM dexamethasone, 0.5 µM 3-isobutyl-1-methylxanthine (Merck Life Science, Madrid, Spain), and 50 µM indomethacin (Merck Life Science, Madrid, Spain). For chondrogenic differentiation, 2.5 × 10^5^ cells were cultured in 15 mL conical tubes with basal medium supplemented with 50 nM L-ascorbic acid, 6.25 µg/mL bovine insulin (Merck Life Science, Madrid, Spain), and 10 ng/mL transforming growth-β factor (TGF-β) (Merck Life Science, Madrid, Spain). After 3 weeks, cells were fixed with 10% formalin solution (Merck Life Science, Madrid, Spain) and stained with Alizarin Red S (Merck Life Science, Madrid, Spain) for osteogenic differentiation, Oil Red O (Merck Life Science, Madrid, Spain) for adipogenic differentiation and Alcian Blue (Merck Life Science, Madrid, Spain) for chondrogenic differentiation.

The results obtained with the staining were validated by quantitative reverse transcription real-time polymerase chain reaction (RT-qPCR). Briefly, samples were collected at the end of each stage of differentiation. RNA was extracted following the manufacturer’s recommendations from the RNeasy Mini Kit (Qiagen, Madrid, Spain). After extraction, total RNA was quantified and for complementary deoxyribonucleic acid (cDNA) synthesis. RT-qPCR was performed with StepOne™ PCR system (Life Technologies, Carlsbad, CA, USA) by using specific TaqMan fluorescent probes listed in the Supplementary Table S1. All samples were assayed in triplicate and normalized based on their constitutive 18 S ribosomal RNA.

#### 2.3.3. IFNγ Licensing by Western Blot

For protein isolation, cells were immersed in ice-cold radioimmunoprecipitation assay (RIPA) Lysis and Extraction Buffer (Thermo Fisher Scientific, Rockford, IL, USA) with Halt Protease inhibitors (Pierce Biotechnology, Rockford, IL, USA). Afterwards, lysates were spun down at 4 °C. Supernatants were collected and protein was quantified by Pierce Bicinchoninic acid (BCA) protein assay kit (Thermo Fisher Scientific, Rockford, IL, USA). Then, 30 mg of protein was mixed with 1/10 parts of dithiothreitol (DTT) (Roche Diagnostics Deutschland GmbH, Mannheim, Germany) and ¼ parts of 4 × Laemmle Sample Buffer (Bio-Rad Laboratories, Hercules, CA, USA). The mixture was heated at 85 °C for 10 min and cooled immediately. Samples were run on 10% Criterion™ Stain-Free™ Precast Gel (Bio-Rad Laboratories, Madrid, Spain) and transferred onto Polyvinylidene fluoride (PVDF) membranes using the Transfer Packs (Bio-Rad Laboratories, Hercules, CA, USA). Anti-IDO antibody (1:1000) (Cell Signaling Technology, Danvers, MA, USA) and anti-β-actin antibody (1: 4000) (Merk Life Science, Madrid, Spain) as a loading control were used, followed by horseradish peroxidase (HRP)-conjugated Goat Anti-Rabbit (Bio-Rad Laboratories, Hercules, CA, USA) and Goat Anti-Mouse (Bio-Rad Laboratories, Hercules, CA, USA) secondary antibodies (1:10,000). Finally, enhanced chemiluminescence (ECL) Substrate (1:1) (Bio-Rad Laboratories, Madrid, Spain) was added and blots were visualized using a multiplex (MP) Imaging System (Bio-Rad Laboratories, Madrid, Spain). Obtained band signals were processed using Image Lab™ Software (4.0.1, Bio-Rad Laboratories, Hercules, CA, USA).

#### 2.3.4. IFNγ Licensing by RT-qPCR

The *IDO* expression was also analyzed at gene expression level by RT-qPCR. The samples were processed as previously described. The used TaqMan fluorescent probes are listed in the Supplementary Table S1.

#### 2.3.5. CD90 Immunohistochemistry

Freshly isolated hair follicles were fixed with 10% formalin for 20 min. Fixed hair follicles were then blocked with 2% bovine albumin serum (Merck Life Science, Madrid, Spain) and 0.5% Triton-X (Merck Life Science, Madrid, Spain) for 2 h at room temperature. After, they were incubated with 6 mg/mL anti-CD90 (Abcam, Amsterdam, The Netherlands) overnight at 4 °C. This was followed by a subsequent incubation with the secondary antibody (Life technologies, Paisley, UK) for 2 h at room temperature. Afterwards, the hair follicles were incubated with 0.001% of 4,6-diamidino-2-phenylindole (DAPI) (Molecular Probes, Eugene, OR, USA). Then, hair follicles were washed and mounted. Images were taken in the Zeiss LSM800 (Jena, Madrid, Spain).

### 2.4. Immunomodulatory Properties of HF-MSCs

#### 2.4.1. PBMC Proliferation Assay

The capacity of MSCs to suppress the proliferation of PBMCs was assessed by means of a Carboxyfluorescein succinimidyl ester (CFSE)-based proliferation assay (Life Technologies Corporation, Eugene, OR, USA). Briefly, PBMCs were labeled with 5 µM CFSE following the manufacturer’s instructions. PBMCs were stimulated with 2.5 µg/mL Concanavalin A (Merck Life Science, Madrid, Spain) in the absence or presence of either MSCs at different ratios for 5 days. At day 5, cells were collected and CFSE intensity was determined by flow cytometry. Data were acquired using the MACSQuant^®^ analyzer (Miltenyi Biotec, Bergisch Gladbach, Germany). Data were analyzed using FlowJo^TM^ v10.6.2 Software (BD Life Sciences, Ashland, OR, USA).

#### 2.4.2. PBMCs Population Change during MSCs Co-Culture

PBMCs were stimulated with 2.5 µg/mL Concanavalin A in the absence or presence of MSCs for 5 days. Then, PBMCs were collected and stained with the following antibodies: Anti-CD3-VioGreen, anti-CD19-PEVio770, anti-CD8-APC (Miltenyi Biotec, Bergisch Gladbach, Germany), or corresponding isotype controls following the manufacturer’s instructions. Data were acquired using the MACSQuant^®^ analyzer (Miltenyi Biotec, Bergisch Gladbach, Germany). Data were analyzed using FlowJo^TM^ v10.6.2 Software (BD Life Sciences, Ashland, OR, USA).

#### 2.4.3. Induction of Regulatory T Cells

For in vitro regulatory T cell induction, CD4+ T lymphocytes were seeded in wells pretreated with 1 μg/mL anti-CD3 (Thermo Fisher Scientific, San Diego, CA, USA) and then stimulated with 25 µL of Dynabeads Human T-Activator CD3/CD28 (Life Technologies AS, Norway), 5 ng/mL TGF-β, and 0.1 μM/mL all-trans-retinoic acid (Merck Life Science, Madrid, Spain) for 2 days with T lymphocytes alone or in the presence of MSCs at a ratio of 1:10 (MSCs: CD4+). After 2 days, cells were stained with the following antibodies: Anti-CD4-VioBlue and anti-CD25-PE or with the corresponding isotype control antibodies following the manufacturer’s instructions (Miltenyi Biotec, Bergisch Gladbach, Germany). Then, cells were fixed and permeabilized using FoxP3 Staining Buffer Set (Miltenyi Biotec, Bergisch Gladbach, Germany). Finally, T lymphocytes were stained with anti-FoxP3-APC or with the corresponding isotype control antibodies. Data were acquired using the MACSQuant^®^ analyzer (Miltenyi Biotec, Bergisch Gladbach, Germany). Data were analyzed using FlowJo^TM^ v10.6.2 Software (BD Life Sciences, Ashland, OR, USA).

#### 2.4.4. Induction of Differentiated Macrophages and Their Co-Culture with HF-MSCs

For in vitro macrophage induction, purified CD14+ monocytes were cultured in a complete RPMI medium (Life Technologies, Bleiswijk, The Netherlands) supplemented with 10% heat-inactivated calf serum (Merck Life Science, Madrid, Spain), 2 mM L-glutamine (Merck Life Science, Madrid, Spain), 1% antibiotic/antimycotic solution, and 100 ng/mL Macrophage Colony-Stimulating Factor (M-CSF) (Miltenyi Biotec, Bergisch Gladbach, Germany) for 8 days. Afterwards, M0 macrophages were polarized towards a M1 phenotype by incubating them with 120 ng/mL of IFNγ and 10 ng/mL of Lipopolysaccharides (LPS) (Merck Life Science, Madrid, Spain) for 48 h. For M2 phenotype, M0 macrophages were incubated with 2 µg/mL IL-4 (Peprotech, London, UK) for 48 h. MSCs were co-cultured with either M0, M1, or M2 macrophages at a ratio of 1:1. After 2 days macrophages were stained with the following antibodies (Miltenyi Biotec, Bergisch Gladbach, Germany): anti-CD163-APC, anti-CD64-APC-Vio770, anti-CD209-PE-Vio770, anti-CD86-FITC, anti-MHC-I- PeVio770, anti-CD206 VioBlue, or with the corresponding isotype control antibodies. Data were acquired by flow cytometry using the MACSQuant^®^ analyzer (Miltenyi Biotec, Bergisch Gladbach, Germany). Data were analyzed using MACSQuantify software (Miltenyi Biotec, Bergisch Gladbach, Germany) and FlowJo^TM^ v10.6.2 Software (BD Life Sciences, Ashland, OR, USA).

### 2.5. Data Analysis and Statistics

The experiments are shown as mean of at least 3 independent experiments ± SD for line and bar graphs. First, the normal distribution of the data was determined using Shapiro-Wilk test. To detect statistical significances between two groups, t-test was performed in normally distributed data. The non-parametric Mann-Whitney U test was used if the data were non-normally distributed. For multiple comparisons, one-way ANOVA was used. In One-way ANOVA, the Bartlett’s or Levene test was performed to determine the homogeneity of variances. If homogeneous, the Tukey or Bonferroni post-hoc test was applied; if not, the Tamhane post-hoc test was used. All statistical analyses were performed by SPSS 23 (IBM SPSS, Chicago, IL, USA) and Prism GraphPad v9 (GraphPad Software, San Diego, CA, USA).

## 3. Results

### 3.1. Characterization of HF-MSCs

First, we characterized the HF-MSCs regarding their (i) localization and neural crest-like phenotype; (ii) “stemness” properties; and (iii) responsiveness to pro-inflammatory stimulus. We explored all these characteristics with cells of passage 2 and passage 10 to detect any possible significant change in the quality of the cells throughout the recommended expansion range.

#### 3.1.1. Isolation, Localization, and Freshly Harvested Phenotype

**Figure 1:**
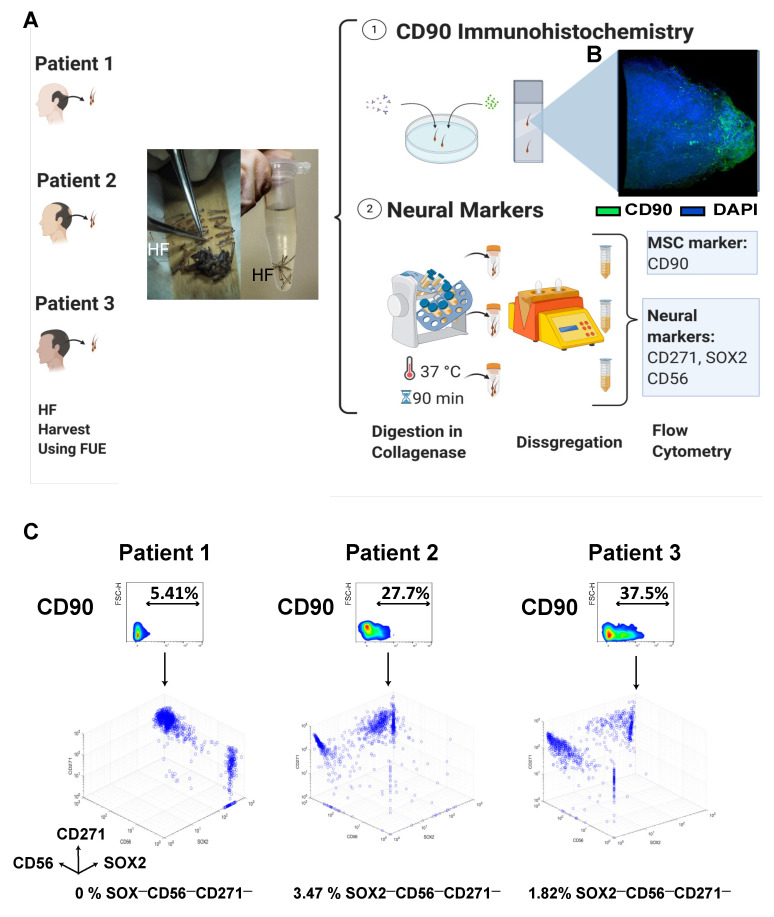
Isolation of HF-MSCs and neural origin characterization of freshly harvested HF-MSCs. (**A**) Hair follicle extraction using the FUE technique from patients undergoing hair transplant therapy. After the extraction, the hair follicles were cleaned and used either for an (1) immunochemistry assay or digested and used for the (2) expression of neural markers (CD271, SOX2, and CD56) in freshly harvested cells. (**B**) Immunohistochemistry of the hair follicle showing the MSC marker CD90 in green and DAPI in blue at 63× amplification. (2) Schematic representation of the procedure to obtain single cell suspension from freshly harvested hair follicles and subsequent staining of the HF-MSCs with neural markers. (**C**) Density plot representation of the CD90 variation with respect to FSC-H for the three different patients and three-dimensional representations of the intensity of neural markers, SOX2 (*x*-axis), CD56 (*y*-axis), and CD271 (*z*-axis) after the CD90 gating. FUE, follicular unit extraction. HF, hair follicle. MSC, mesenchymal stromal cells.

Hair follicles from patients aged between 25–40 were harvested using the Follicular Unit Extraction (FUE) technique and used for either immunohistochemical staining or flow cytometry analysis (Figure 1A). With the aim of studying the exact localization of our cells within the hair follicle, we conducted a CD90 immunohistochemistry. The immunohistochemistry showed that CD90+ cells were found only in the lower part of the hair follicles where the dermal papilla and dermal cup/lower dermal sheath are located (Figure 1B, Video S1).

One of the main characteristics of the cells whose origin is the dermal papilla is their possible neural origin. To confirm that our cells expressed neural markers that could suggest their neural crest origin, we dissociated the hair follicles and conducted flow cytometry for three neural markers: CD271, SOX2, and CD56. The histograms showed remarkable differences in the percentages of neural marker expression between each patient, indicating the inter-patient variability (Figure 1C, Figure S1). However, as noticed in the three-dimensional (3D) density plot, at least 96% of CD90+ cells were also positive for at least one of the markers. Of note, once the cells were cultured in vitro, CD271 expression decreased rapidly, whereas CD56 and SOX-2 were gradually lost over subsequent cell passages (Figure S2).

Based on the results of these two assays, we suggested that these cells could come from the dermal sheath.

#### 3.1.2. “Stemness” Characterization

**Figure 2:**
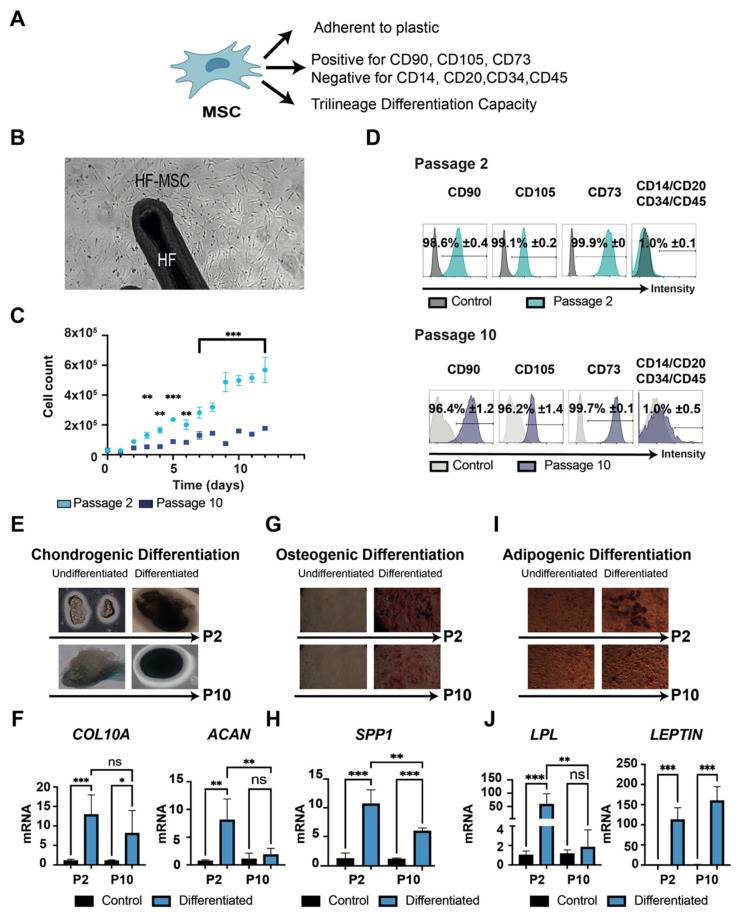
Characterization of minimal criteria to define MSCs. (**A**) A schematic representation of the MSCs and their necessary properties. (**B**) Microscopic image representing the capacity of the HF-MSCs to adhere to plastic at 10× amplification. (**C**) Graphical representation of the proliferation potential of HF-MSCs at both passage 2 and passage 10. (**D**) Cells cultured at passage 2 and passage 10 were harvested and labelled with antibodies against the following cell surface proteins: CD90, CD105, CD73, CD14, CD20, CD34, and CD45. Flow cytometry histograms of cells at P2 (teal) and P10 (dark blue) are shown. Gray histograms indicate isotype control for each antibody. Each datapoint represents the mean for three wells ± SD. (**E**–**J**) Trilineage differentiation of cells at both passage 2 and passage 10. (**E**) Chondrogenic differentiation capacity of HF-MSC after 21 days with the differentiation medium. Blue color represents the secretion of sulfated proteoglycans visualized with Alcian blue at 10× amplification. (**F**) Chondrogenic differentiation was further confirmed by *COL10A* and *ACAN* gene expression. (**G**) Osteogenic differentiation capacity of HF-MSCs. Deposition of calcified nodules was visualized by Alizarin Red staining at 10× amplification. (**H**) Osteogenic differentiation was further confirmed by *SPP1* gene expression. (**I**) Adipogenic differentiation capacity of HF-MSCs. Red color indicates the staining of lipid vesicle-forming adipocytes by Oil Red staining at 20× amplification. (**J**) Adipogenic differentiation was further confirmed by *LPL* and *LEPTIN* gene expression. Each datapoint represents the mean for at least three wells ± SD. Statistical significance: *** *p* < 0.001, ** *p* < 0.01, * *p* < 0.05, ns, no significant difference *p* > 0.05. MSC, mesenchymal stromal cells. P2, passage 2. P10, passage 10. HF-MSC, hair follicle-derived mesenchymal stromal cell.

HF-MSCs were then characterized according to the 2006 International Society for Cellular Therapy (ISCT) criteria (Figure 2A) [24] at both passages. HF-MSCs displayed a high capacity to adhere to plastic and to proliferate (Figure 2B). Similar to MSCs from other tissues, the attached cells showed a significant decrease in proliferation rate capacity when cells reached passage 10 (Figure 2C) [25,26]. Subsequently, both passages met the criteria regarding their phenotype, with non-significant differences in expression levels of the explored markers between MSCs from different passages (Figure 2D). Finally, to demonstrate the differentiation potential of HF-MSCs, both passages were cultured under the conditions to induce chondrogenic, osteogenic, and adipogenic differentiation (Figure 2E–J, Figure S3). After 3 weeks of culture, chondrogenic differentiation was confirmed in both passages by Alcian blue staining and the expression of *COL10A* and *ACAN* genes. Both genes were highly expressed in passage 2—*p* < 0.001 and *p* < 0.01 respectively compared with control—but only *COL10A* was expressed by cells at passage 10—*p* < 0.05 compared with control—showing non-statistical differences against passage 2 (Figure 2F). Differentiation into the osteogenic lineage was shown by Alizarin Red staining and the gene expression of *SPP1* (Figure 2G–H). *SPP1* was highly expressed in passage 2 cells—*p* < 0.001 compared with control—but there was reduced expression in passage 10 cells compared with passage 2—*p* < 0.01, although expression was still significantly higher than the control—*p* < 0.001. Adipogenic differentiation was evaluated by Oil Red O staining and the expression of *LPL* and *LEPTIN* genes (Figure 2I–J). *LEPTIN* was highly expressed at both passages—*p* < 0.001 compared with control—with no significant difference between passages—*p* > 0.05. However, the expression of *LPL* was only detected at passage 2—*p* < 0.001 compared with control and *p* < 0.01 compared with passage 10. These results suggested that HF-MSCs fulfilled all commonly considered attributes of bona fide mesenchymal stem cells, but also that the cells gradually lost stemness properties in adaptation to culture.

#### 3.1.3. Immunomodulatory Responsiveness and Maintenance of Immunoevasiveness against Pro-Inflammatory Stimulus

**Figure 3:**
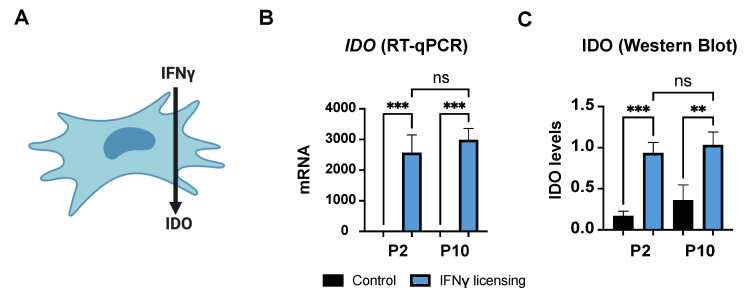
Effect of IFNγ on HF-MSCs. (**A**) IFNγ induces expression of IDO in human MSCs. (**B**,**C**) Representation of *IDO* gene expression levels in HF-MSCs by (**B**) qRT-PCR and IDO protein levels in HF-MSCs by (**C**) Western Blot. Each datapoint represents the mean for at least three wells ± SD. Statistical significance: *** *p* < 0.001 ** *p* < 0.01, ns, no significant difference *p* > 0.05. IFNγ interferon gamma. IDO, indoleamine 2,3-dioxygenase. P2, passage 2. P10, passage 10.

MSCs can secrete IDO, among other immunomodulatory molecules, in response to pro-inflammatory stimuli, which further modulates the phenotype of immune cells such as macrophages and T cells (Figure 3A). Thus, we examined if HF-MSCs were able to produce IDO in response to a pro-inflammatory signal. IFNγ-treated HF-MSCs exhibited significantly higher IDO expression compared with the untreated cells at both the mRNA—*p* < 0.001 each passage compared with its control—and protein levels—*p* < 0.001 passage 2 compared with its control, and *p* < 0.01 passage 10 compared with its control—with no statistical differences (*p* > 0.05) between passages (Figure 3B,C and Figure S4).

**Figure 4:**
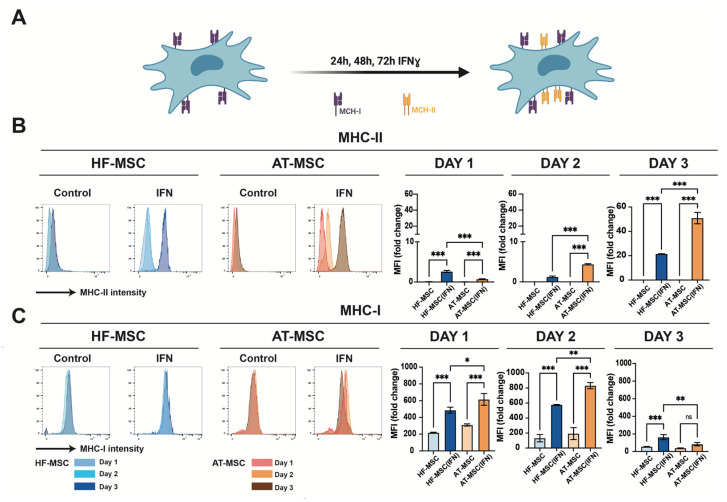
Effect of IFNγ on HF-MSCs immunoevasiveness. (**A**) IFNγ upregulates the MHC-II expression in MSCs. (**B**,**C**) Histograms from flow cytometry assays depicting percentage of HF-MSCs positively stained for MHC-II (**B**) and MHC-I (**C**) on unstimulated and IFNγ-licensed HF-MSCs and AT-MSCs over 1, 2, or 3 days. Each datapoint represents the mean for at least three biological replicates ± SD. Statistical significance: *** *p* < 0.001 ** *p* < 0.01, * *p* < 0.05, ns, no significant difference *p* > 0.05. IFNγ, interferon gamma. MHC-I, major histocompatibility class I molecule. MHC-II, major histocompatibility class II molecule. HF-MSCs, hair follicle-derived MSCs. AT-MSC, adipose tissue-derived MSCs.

It is known that IFNγ induces expression of MHC-II in MSCs (Figure 4A). To assess if this too influences our cells, the expression of MHC-II and MHC-I was measured when HF-MSC and AT-MSCs were licensed with IFNγ over 1, 2, or 3 days. During the first two days, HF-MSCs showed significantly lower expressions of both MHC-I and MHC-II in comparison with AT-MSCs (Figure 4B,C). In regard to MHC-I, there was an expression peak at day 2 for AT-MSCs, and at day 3 for HF-MSCs. MSCs have been previously described to have the ability to auto-downregulate the surface expression of MHC-I, leaving the cell with low immunity in presence of IFNγ [27]. However, in the case of AT-MSCs, at day 3 this low immunity could be compromised due to the high MHC-II expression. On the contrary, HF-MSCs showed significantly lower expression of MHC-II—*p* < 0.001 compared with AT-MSCs—indicating that HF-MSCs may remain immunoevasive even when exposed to a proinflammatory environment.

### 3.2. Immunomodulatory Potential of HF-MSCs

Having confirmed the “stemness” of the HF-MSCs and their responsiveness to pro-inflammatory cues, we next addressed the extent of their immunomodulatory potential.

#### 3.2.1. Immunosuppressive Potential

**Figure 5:**
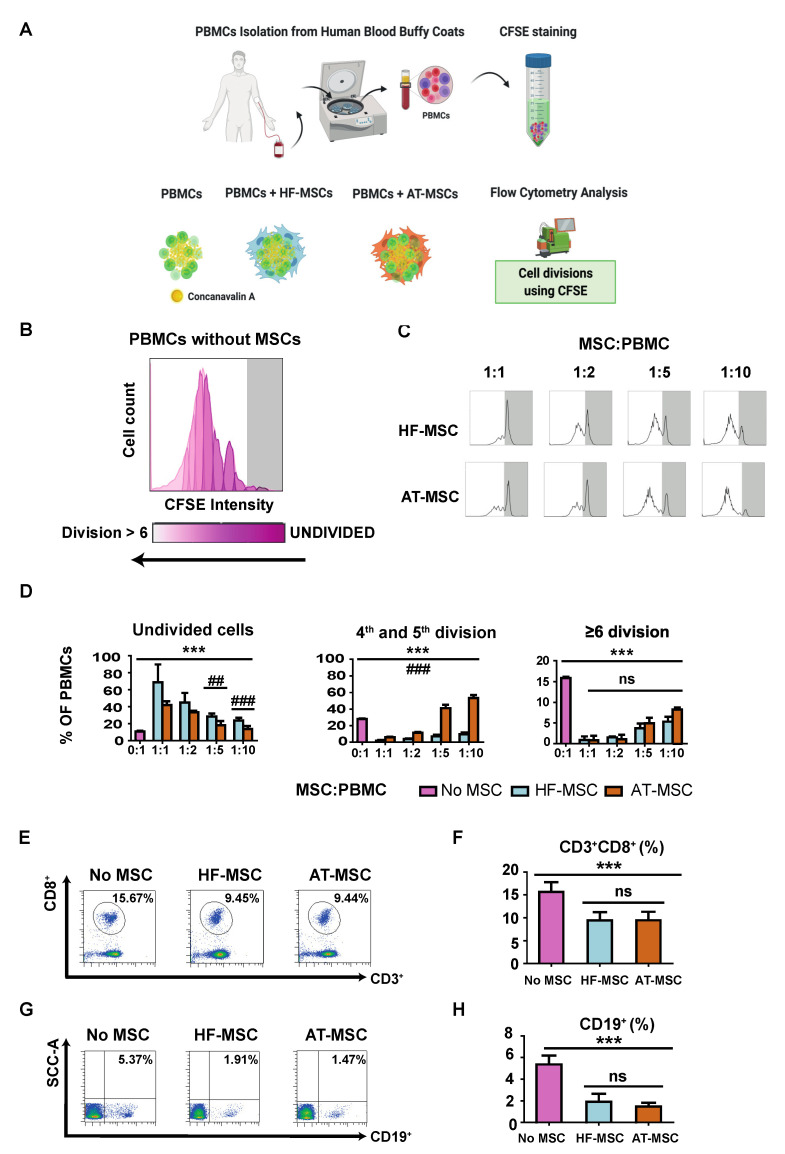
Immunosuppressive effect of MSCs in concanavalin-A stimulated PBMCs. (**A**) PBMCs were isolated from the blood of human healthy donors and stained with CFSE to monitor their proliferation. (**B**,**C**) Flow cytometry histograms of the proliferation profile of PBMCs alone or co-cultured with MSCs. (**D**) Percentages of PBMCs remaining undivided, undergoing four and five divisions, and undergoing more than or six divisions. (**E**,**G**) Flow cytometry dot plots depicting the populations of CD3+CD8+ T cells (**E**) and CD19 cells (**G**) and their percentages (**F**,**H**) when PBMCs were cultured alone or with MSCs. Each datapoint represents the mean for at least three wells ± SD. Statistical significance: *** *p* < 0.001, ns, no significant difference *p* > 0.05 when compared with PBMCs without MSCs; ### *p* < 0.001 and ## *p* < 0.01 when comparing HF-MSCs with AT-MSCs. ns: not significant differences, *p* > 0.05. PBMCs, peripheral blood mononuclear cells. HF-MSCs, hair follicle-derived MSCs. AT-MSCs, adipose tissue-derived MSCs.

Initially, we analyzed the capacity of HF-MSCs to inhibit the proliferation of PBMCs (Figure 5). PBMCs were cultured alone or in the presence of either HF-MSCs or AT-MSCs at different MSC:PBMCs ratios—1:1, 1:2, 1:5, and 1:10—for 5 days in the presence of Concanavalin A to stimulate the PBMCs (Figure 5A). PBMCs alone showed a strong proliferative response (Figure 5B,D). In contrast, PBMCs demonstrated a significantly decreased proliferation when co-cultured with both MSCs in a dose-dependent manner—*p* < 0.001—(Figure 5C,D). HF-MSCs stimulated considerably a higher percentage of undivided cells for 1:5—*p* < 0.01—and 1:10—*p* < 0.001—ratios, and a lower percentage of cells between four and five divisions—*p* < 0.001—compared with AT-MSCs. In addition, HF-MSCs pretreated with IFNγ did not elicit a stronger inhibitory response on PBMC proliferation (Figure S5). Finally, MSCs were able to decrease the percentage of cytotoxic CD3+CD8+ T cells (Figure 5E,F) and CD19+B cells (*p* < 0.001) (Figure 5G,H).

#### 3.2.2. Capacity to Generate Regulatory T Cells

**Figure 6:**
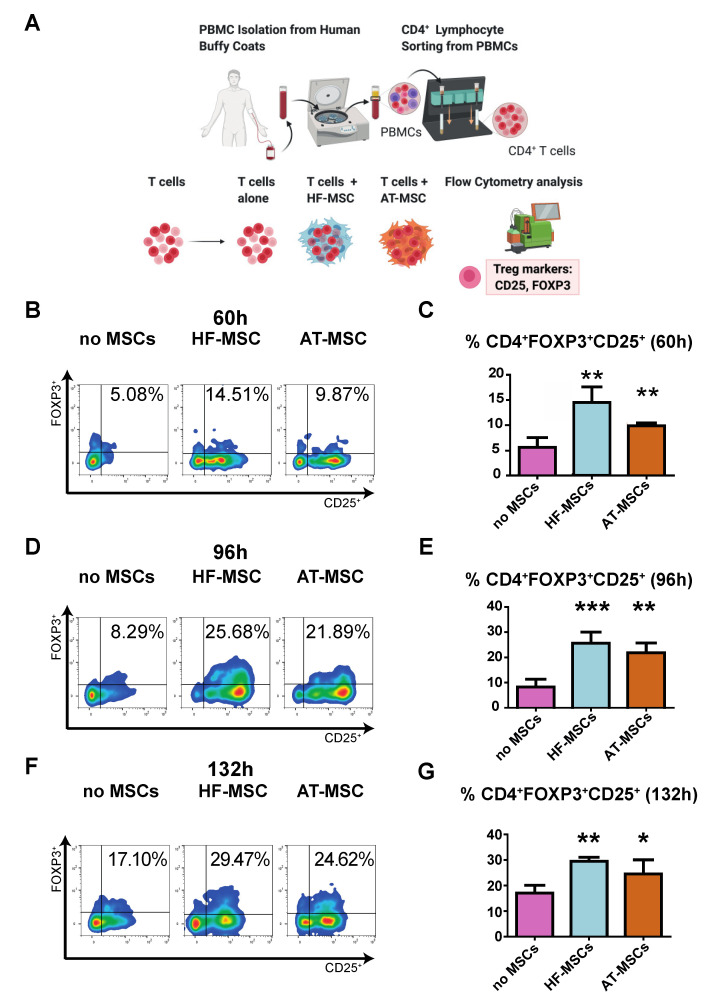
Effect of MSCs on induction of CD4+CD25+FOXP3+ T regulatory cells. (**A**) Schematic of the study. (**B**–**G**) Dot plots (**B**,**D**,**F**) and their bar-graph representations (**C**,**E**,**G**) exhibiting the percentages of T regs when cultured alone or with MSCs over 60 h (**B**,**C**), 96 h (**D**,**E**) and 132 h (**F**,**G**). Each data point represents the mean for at least three wells ± SD. Statistical significance: *** *p* < 0.001, ** *p* < 0.01, and * *p* < 0.05 when compared against the control group without MSCs. PBMCs, peripheral blood mononuclear cells. HF-MSCs, hair follicle-derived MSCs. AT-MSCs, adipose tissue-derived MSCs.

We next evaluated the capacity of HF-MSCs to induce T regulatory differentiation as compared to AT-MSCs. To this end, CD4+ T cells were co-cultured with or without MSCs for 60 h, 96 h, and 132 h and the number of cells exhibiting a T regulatory phenotype—CD4+CD25+FOXP3+—was analyzed by flow cytometry (Figure 6). There was a significant increase in T regulatory induction at all assayed time points when T cells were cocultured with MSCs, with peak induction at 96 h—*p* < 0.001 and *p* < 0.01 for HF-MSCs and AT-MSCs vs. the control group, respectively.

#### 3.2.3. Capacity to Regulate Macrophage Phenotype

**Figure 7:**
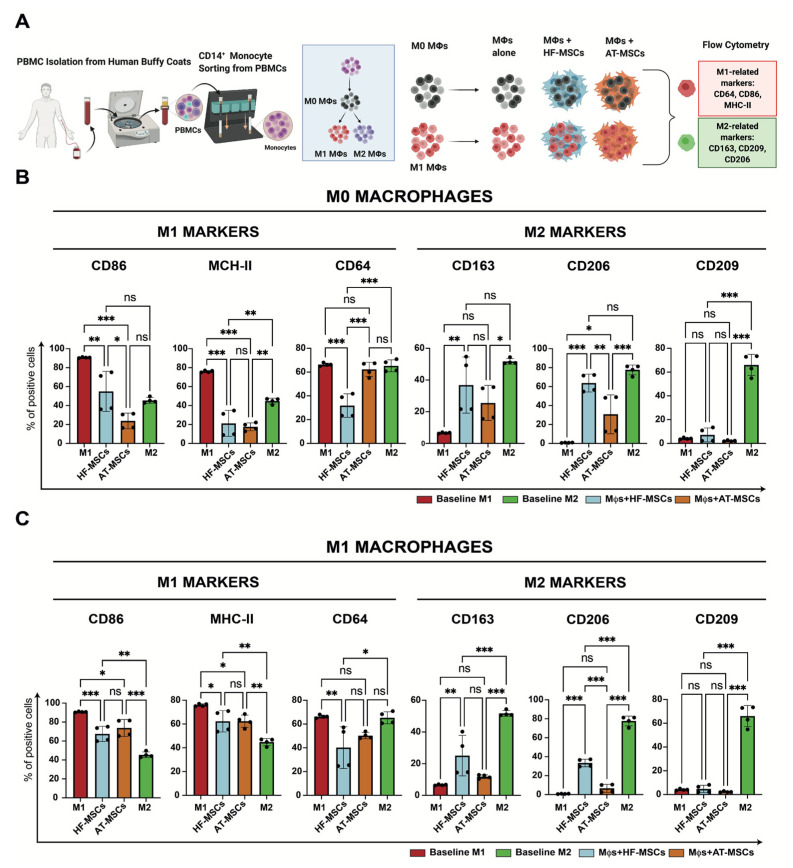
Effect of MSCs on the modulation of macrophage phenotype. (**A**) Schematic of the study. (**B**,**C**) % of cells positive for either M1-related or M2-related marker expression when M0 macrophages (**B**) or M1 macrophages (**C**) were cocultured with MSCs. Each datapoint represents the mean ± SD of four wells. Statistical significance: *** *p* < 0.001 and ** *p* < 0.01 and * *p* < 0.05 when compared with macrophages without MSCs; ns: not significant differences, *p* > 0.05. PBMCs, peripheral blood mononuclear cells. Mφ, macrophages. HF-MSCs, hair follicle-derived MSCs. AT-MSCs, adipose tissue-derived MSCs.

Based on the observation that MSCs promote M2 macrophage polarization, we next investigated the effects of HF-MSCs and AT-MSCs on macrophages (Figure 7 and Figures S6–S8). Regarding M1 markers, macrophages co-cultured with HF-MSCs and AT-MSCs showed significantly lower expression levels of CD86—*p* < 0.01 and *p* < 0.001, respectively—and MHC-II—*p* < 0.001 and *p* < 0.001, respectively—than the M1 control group, reaching values like or even lower than the M2 control. HF-MSCs but not AT-MSCs caused macrophages to express lower levels of CD64 compared with the M1 control. For M2 markers, only HF-MSCs were able to express CD206 and CD163 to the same extent as the positive control M2—*p* > 0.05, while AT-MSCs were not able to express these markers as much, exhibiting significant differences between them and the positive M2 control—*p* < 0.05 for CD206 and *p* < 0.001 for CD163.

To determine whether the macrophage pre-polarization state has an impact in the crosstalk, macrophages were further polarized towards M1 and M2 phenotypes (Figure 7A, Figure S7). Overall, when M1 macrophages were cocultured with the MSCs, they downregulated M1 markers and upregulated M2 markers (Figure 7C). Specifically, HF-MSCs and AT-MSCs were able to lower the expression of the M1 markers CD86—*p* < 0.001 and *p* < 0.05 respectively—and HLA-DR—*p* < 0.001 and *p* < 0.001, respectively—as compared with the M1 control group. No major differences were observed in the downregulation of M1 markers between HF-MSC and AT-MSC groups, although the expression of CD64 was decreased to a greater extent by HF-MSCs than by AT-MSCs—*p* < 0.05 for the HF-MSC group and *p* > 0.05 compared with the M1 control. With respect to M2 markers, co-culture with HF-MSCs caused macrophages to upregulate CD163 and CD206—*p* < 0.01.

Furthermore, the addition of HF-MSCs or AT-MSCs did not significantly alter M2 macrophages (Figure S7). Additionally, gene expression analysis validated that ability of the HF-MSCs to skew macrophage phenotype towards an M2 phenotype (Figure S8).

## 4. Discussion

In this study, we isolated and characterized MSCs from the human hair follicle. HF-MSCs showed immunomodulatory potential equal to or exceeding that of AT-MSCs, including inhibition of PBMC proliferation, suppression of cytotoxic T cell induction, promotion of regulatory T cell differentiation, and modulation of macrophage phenotype from M1 to M2. These results suggest that HF-MSCs should be considered a viable alternative to BM- and AT-MSCs for therapies that rely on their immunomodulatory potential.

Bone marrow and adipose tissue are the most studied sources of MSCs in clinical trials, with both being used with almost equal frequency [10]. However, there are substantial limitations in using these cells, mostly regarding the harvesting procedure and that have shown signs of senescence early during expansion [28]. Specifically, AT-MSCs are limited by the need to overcome certain challenges such as the extraction technique invasiveness and extraction limitations. In contrast, HF-MSCs can be obtained by means of a relatively painless, less invasive, and low-risk harvesting procedure, which also presents a lower risk of contamination.

The cells analyzed throughout this study came specifically from the hair follicle lower dermal sheath/dermal papilla, and these freshly isolated cells expressed the neural markers SOX2, CD271, and CD56. CD271 has been proposed as one of the most specific marker for the characterization and purification of different origin human mesenchymal stromal cells [29]. Interestingly, CD271 expression is found in cells within human neural crest-derived tissues [30,31]. As the hair follicle has many different stem cell niches to focus on, an immunohistochemistry analysis was conducted to ensure that the cells that were being analyzed came from the mesenchymal niche of the hair. In concordance of previous studies, most of the stained section were in the lower part of the hair follicle, where the dermal papilla/dermal cup is localized [32,33,34]. In addition to the dermal papilla, the mesenchyme of the follicle is also composed of a follicle smooth muscle known as the dermal sheath [35]. Work conducted by Rahmani et al. showed the existence of dermal stem cells that are located in the hair follicle dermal sheath [36]. They studied the crosstalk between the dermal sheath and dermal papilla, and they demonstrated the importance of these stem cells in the hair follicle regeneration. Some studies have suggested that a difference between HF-MSCs and other origin MSCs is the possible neural origin of the former one [15,37].

Stemness—strictly interpreted from the minimum criteria defined by the ISCT—and the immunomodulatory responsiveness capacity were maintained even in passage 10. This could be an important feature as it has been described that MSCs lose their differentiation and immunomodulatory capacity earlier than passage 10 [25,26]. HF-MSCs could be cultured until the needed yield is obtained without any concern of losing important characterization MSC qualities.

It is known that this immunosuppressive potential of MSCs is not constitutive, but dependent on the inflammatory environment to which MSCs are exposed [38,39]. For example, it has been demonstrated that pretreatment of MSCs with IFNγ induces a robust expression of IDO, leading to subsequent inhibition of immune cell proliferation [40]. HF-MSCs showed a good responsiveness to pro-inflammatory stimulus, indicating that they did not lose their IDO responsiveness even when cultured for a long period in vitro.

An important characteristic of the MSCs is their immune evasiveness. One of the main reasons for this has been associated with the fact that they do not express MHC-II [41]. However, it has been described that in inflammatory environments, MSCs of diverse origins start expressing the MHC-II molecule, losing their characteristic evasiveness [42]. Importantly, we observed that HF-MSCs expressed significantly lower MHC-II, even when exposed to pro-inflammatory environments, compared with AT-MSCs. This could be due to the hair being an immune-privileged organ, thus deviating the immune response to favor tolerance and suppress immune-mediated inflammation [43,44]. Thus, considering the limited number of cells available for autologous use, HF-MSCs may be a suitable allogenic alternative for MSC-based cell therapy.

HF-MSCs suppressed the proliferation of PBMCs and reduced the populations of CD8+ T lymphocytes and CD19+ B while prompting the regulatory T cell activation at same extent as AT-MSCs. Interestingly, HF-MSCs exerted a more powerful inhibition than AT-MSCs when co-cultured at lower MSC:PBMC ratios. The ability of HF-MSCs to prompt T regulatory differentiation, and to inhibit the response of activated B and T cells, makes this cell type a promising candidate for different immune-mediated diseases where the adaptive immunity plays a critical role.

MSCs have been reported to be able to influence M2 polarization of macrophages, which is an important mechanism in resolution of inflammation [45,46]. A recent study has shown that MSCs located in the outer root sheath of the hair follicle suppressed secretion of proinflammatory cytokines while stimulating the production of anti-inflammatory cytokine IL-10 and the expression of the M2 marker CD163 in M1 macrophages [23]. Our findings that HF-MSCs promoted M2 polarization of primary human macrophages are in accordance with the work published by de Witte et al. and Cutler et al., who showed that MSCs induced expression of the M2 markers CD163 and CD206 on monocytes [47,48]. Interestingly, when directly compared with AT-MSCs, HF-MSCs demonstrated a slightly superior performance. Although both MSC types caused downregulation of M1 markers, only HF-MSCs caused upregulation of any M2 markers in the M1 macrophages. This is of great importance in chronic inflammatory conditions, as a transition from the pro-inflammatory phenotype M1 to the regenerative phenotype M2 is pivotal to overcome the pathological state and heal. A failure in this transition can cause poor healing, as in the case of chronic wounds [49]. Indeed, recent studies have highlighted the importance of MSCs to modulate the macrophage phenotype from M0 and M1 to M2 to kickstart the wound healing process [45,50,51,52,53]. In this study, we noticed that the addition of HF-MSCs resulted in macrophages moving towards an M2 phenotype. This may suggest generation of a hybrid M1/M2 phenotype, which was recently shown to be associated with the production of less fibrotic extracellular matrix compared with a more predominant M2 macrophage phenotype [54].

Future studies with these cells are warranted to find the unique mechanistic pathways that produce such an immunomodulatory effect, especially focusing on both the soluble factors and extracellular vesicles of their secretome. In fact, in recent years, the important role of the secretome in the immunomodulatory therapeutic activity of MSCs has been described, which also represents the basis of cell-free therapies.

## 5. Conclusions

In summary, the cells obtained from the lower dermal sheath/dermal papilla of human hair follicles expressed specific neural markers such as CD56, CD271, and SOX-2 upon extraction, which disappeared at different rates upon culture. In addition, HF-MSCs maintained sufficient stemness properties for at least 10 passages, including standard phenotypic markers, trilineage differentiation, and proliferative capacity. These cells showed responsiveness to the pro-inflammatory cytokine IFNγ while retaining their immuno-privileged status, which makes allogenic HF-MSCs a feasible alternative source of stem cells. In addition, HF-MSC exhibited immunomodulatory properties comparable to or exceeding those of AT-MSCs with respect to crosstalk with cells of both the innate and adaptive immune systems. Together with the advantages of HF-MSC, such as easy accessibility, relatively painless procedures for donors, and lower risk of possible infections, these results suggested that HF-MSCs may be a suitable alternative to AT-MSCs for the treatment of inflammatory disorders. Further studies will fully evaluate the immunogenic properties and immunomodulatory function of HF-MSCs in vivo.

## Data Availability

The data are available from the authors upon request.

## Supplementary Materials

The following supporting information can be downloaded at: https://www.mdpi.com/article/10.3390/biomedicines10020253/s1, Figure S1: Neural origin characterization of freshly harvested HF-MSCs; Table S1: PCR primers for RT-qPCR; Video S1: Immunohistochemistry of the hair follicle showing the MSC marker CD90 in green and DAPI in blue; Figure S2: Specific neural marker expression disappeared when HF-MSCs were cultured in vitro; Figure S3: Trilineage differentiation capacity of HF-MSC at passage 2 and passage 10; Figure S4: Western Blot bands; Figure S5: Immunomodulatory effects of IFN-licensed HF-MSCs in Concanavalin A- stimulated PBMCs; Figure S6: Flow cytometry histograms showing either M2-related phenotypic marker (CD163, CD206 and CD209) or M1-related phenotypic marker (CD86, CD64 and MHC-II) expression; Figure S7: Effect of HF-MSCs and AT-MSCs on the modulation of M2 macrophage phenotype; Figure S8: Effect of HF-MSCs on the gene expression of M0 and M1 macrophages.
