# Supplementary material for: Human Hair Follicle-Derived Mesenchymal Stromal Cells from the Lower Dermal Sheath as a Competitive Alternative for Immunomodulation
Source: Biomedicines. 2022 Jan 24;10(2):253. doi: 10.3390/biomedicines10020253 (PMC8868584; doi:10.3390/biomedicines10020253)
Supplement: Supplementary file 1 [file biomedicines-10-00253-s001.zip › Supplemental Table and Figures.pdf]

Supplemental Table and Figures

Table S1: PCR primers for RT-qPCR

Gene symbol	Gene Name	Prime ID
IDO	*Indoleamine 2,3-dioxygenase 1*	Hs00984148_m1
LPL	*Lipoprotein lipase*	Hs00173425_m1
Leptin	*Leptin*	Hs00174877_m1
ACAN	*Aggrecan*	Hs00153936_m1
COL10A	*Collagen Type X Alpha 1 Chain*	Hs00166657_m1
SPP1	*Osteopontin*	Hs00959010_m1
CXCL11	*C-X-C motif chemokine ligand 11*	Hs00171138_m1
CCL19	*C-C Motif Chemokine ligand 19*	Hs00171149_m1
CXCL10	*C-X-C motif chemokine ligand 10*	Hs00171042_m1
CCL13	*C-C Motif Chemokine ligand 13*	Hs00234646_m1
MRC	*Mannose receptor c-type*	Hs00267207_m1
FN1	*Fibronectin 1*	Hs203717_m1
FGL2	*Fibrinogen like 2*	Hs00173847_m1
CCR7	*C-C chemokine receptor type 7*	Hs01013469_m1
18S	*18S ribosomal RNA*	Hs.PT.39a.22214856

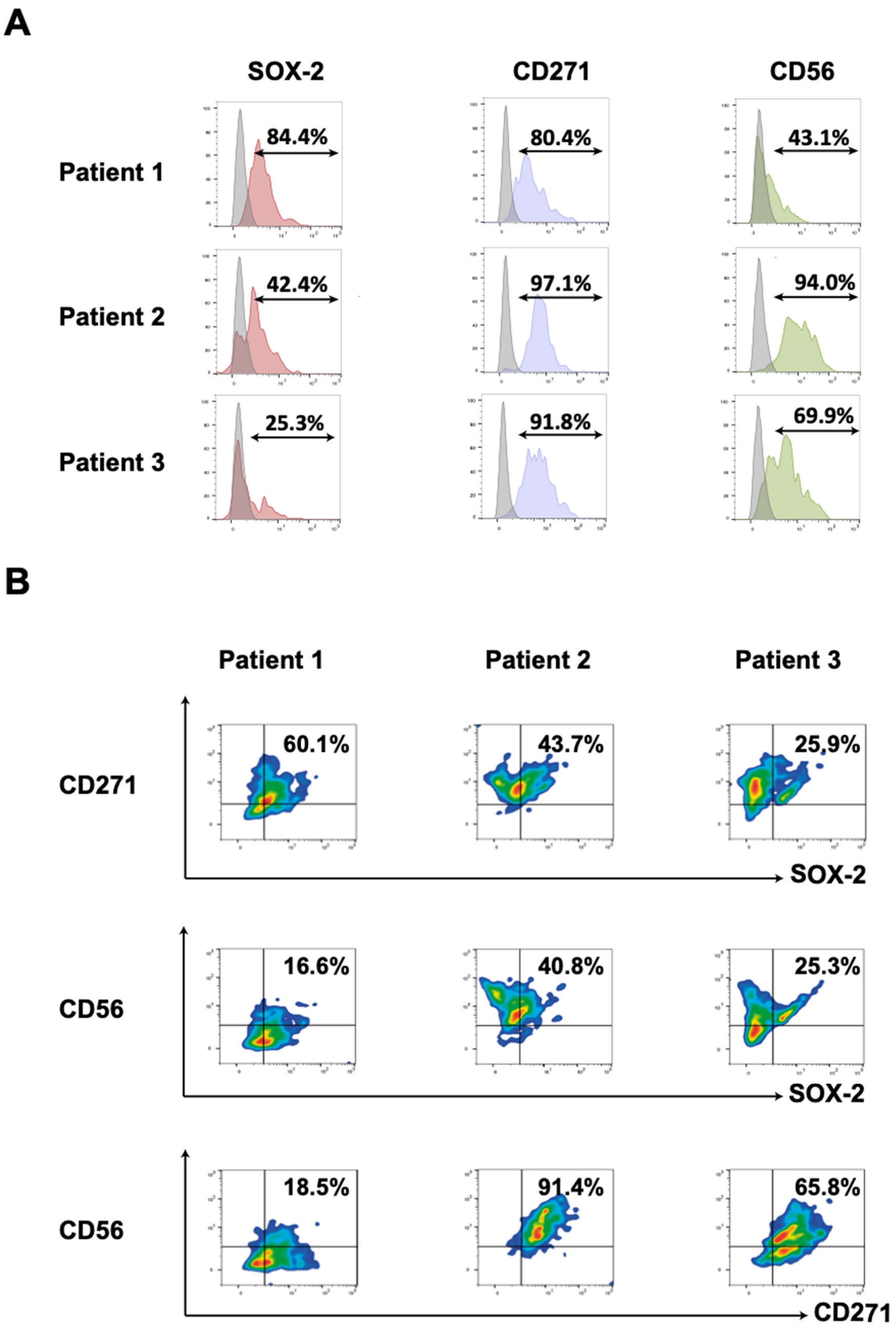
A
SOX-2
CD271
CD56
Patient 1
84.4%
80.4%
43.1%
Patient 2
42.4%
97.1%
94.0%
Patient 3
25.3%
91.8%
69.9%
B
Patient 1
Patient 2
Patient 3
CD271
60.1%
43.7%
25.9%
SOX-2
CD56
16.6%
40.8%
25.3%
SOX-2
CD56
18.5%
91.4%
65.8%
CD271

Figure S1: Neural origin characterization of freshly harvested HF-MSCs. (A) Flow cytometry histograms representing the % of positive CD90+ HF-MSCs expressing SOX2, CD271 or CD56. (B) Flow cytometry density plots representing the percentage of positive cells co-expressing two of the neural markers.

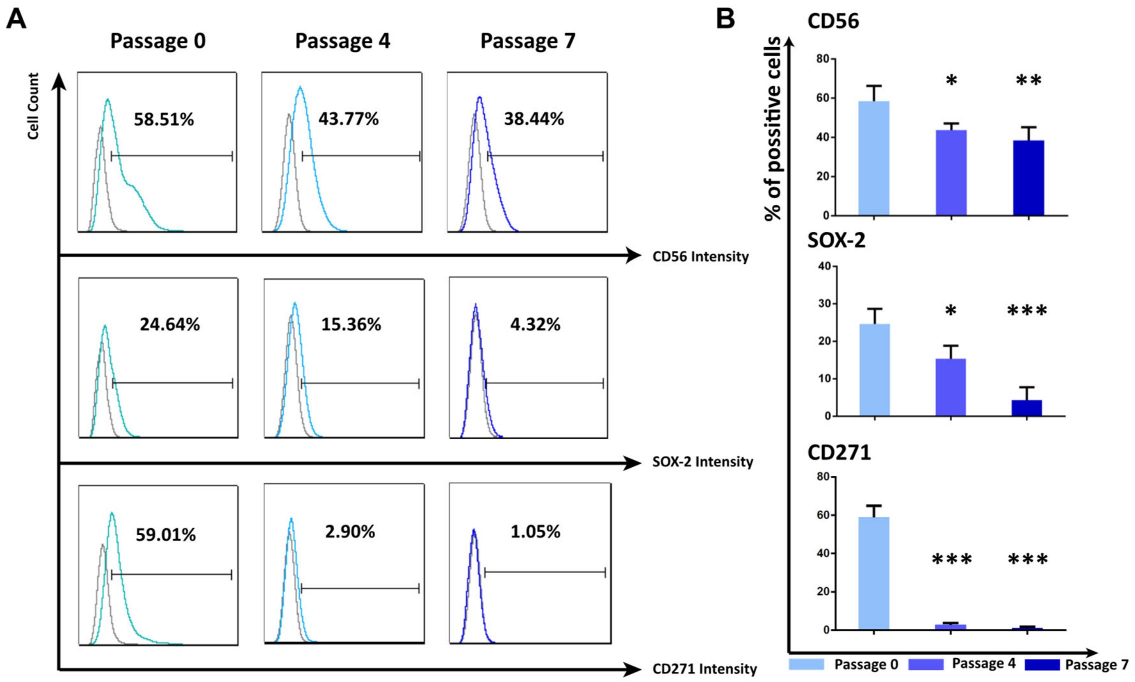

Figure S2: Specific neural marker expression disappeared when HF-MSCs were cultured in vitro. (A) Flow cytometry histograms representing the expression of CD56, SOX-2 and CD271 markers in HF-MSCs of passage 0, passage 4 and passage 7. (B) Graphical bars showing the percentage of positive HF-MSCs to CD56, SOX-2 or CD271 markers in passage 0, passage 4 or passage 7. Each data represents the mean for at least three wells ± SD. Statistical significance: ***p < 0.001, ** p < 0.01 and * p < 0.05 when compared HF-MSCs in passage 4 and passage 7 to passage 0.

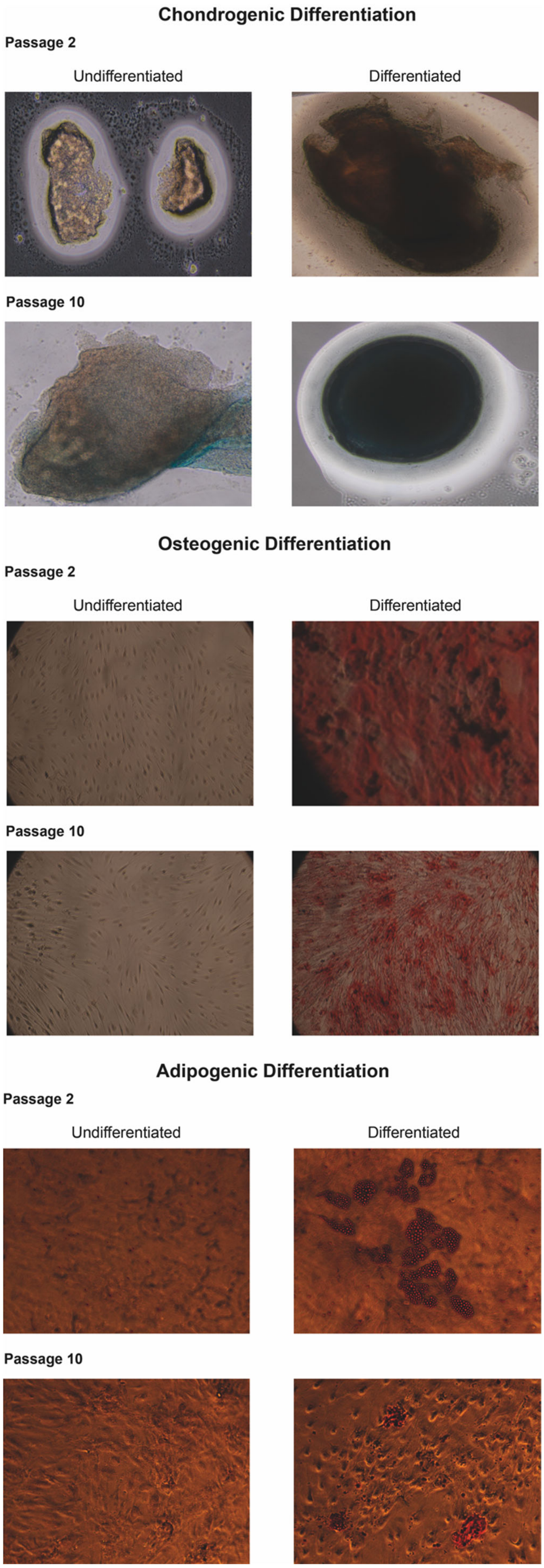
Chondrogenic Differentiation
Passage 2
Undifferentiated
Differentiated
Passage 10
Osteogenic Differentiation
Passage 2
Undifferentiated
Differentiated
Passage 10
Adipogenic Differentiation
Passage 2
Undifferentiated
Differentiated
Passage 10

Figure S3: Trilineage differentiation capacity of HF-MSCs at passage 2 and passage 10. Chondrogenic differentiation capacity of HF-MSC after 21 days with the differentiation medium. Blue color represents the secretion of sulfated proteoglycans visualized with Alcian blue at 10x amplification. Osteogenic differentiation capacity of HF-MSCs. Deposition of calcified nodules was visualized by Alizarin Red staining at 10x amplification. Adipogenic differentiation capacity of HF-MSCs. Red color indicates the staining of lipid vesicle-forming adipocytes by Oil Red staining at 20x amplification.

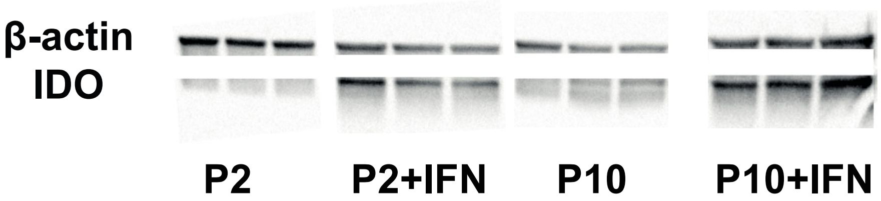

Figure S4: Western Blot bands showing the expression of indolamine 2,3-dioxygenase (IDO) and β-actin when hair follicle mesenchymal stromal cells (HF-MSCs) of passage 2 (P2) and passage 10 (P10) were licensed with interferon (IFN) (P2+IFN, P10+IFN) or without IFN licensing (P2, P10).

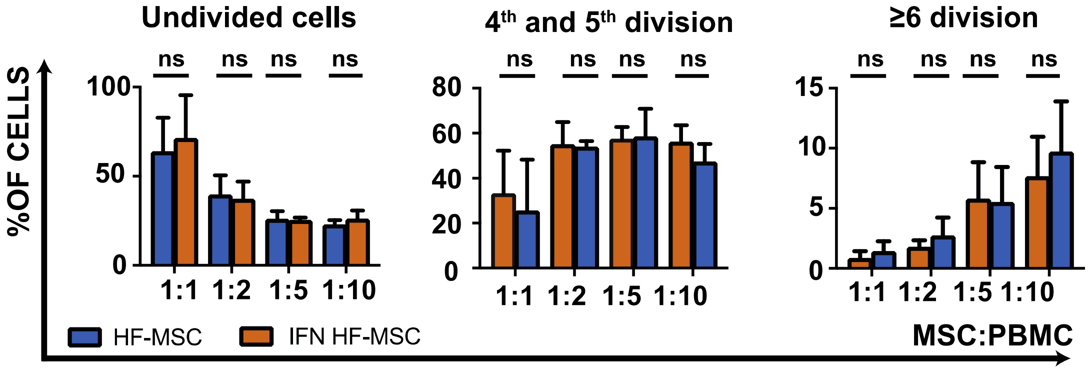

Figure S5: Immunomodulatory effects of IFN-licensed HF-MSCs in Concanavalin A-stimulated PBMCs. (A) The proliferation profiles of the PBMCs after 5 days of co-culture with different ratios (1:1, 1:2, 1:5, 1:10) of IFN-licensed HF-MSCs by means of percentages of PBMCs remaining undivided, undergoing 4 and 5 division, and undergoing more than or 6 divisions. Each data represents the mean for at least three wells ± SD. Statistical significance: ns: no significant differences, $p > 0.05$. PBMCs, peripheral blood mononuclear cells. HF-MSCs, hair follicle-derived mesenchymal stromal cells.

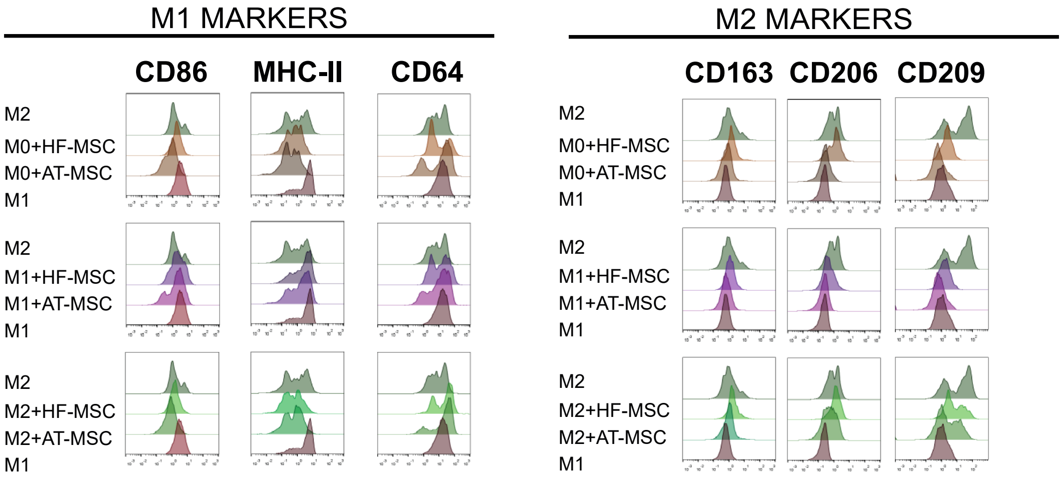

Figure S6: Flow cytometry histograms showing M2-related phenotypic marker (CD163, CD206 and CD209) and M1-related phenotypic markers(CD86, CD64 and MHC-II) expression when M0, M1 or M2 macrophages were co-cultured with HF-MSCs or AT-MSCs. HF-MSCs, hair follicle-derived mesenchymal stromal cells. AT-MSCs, adipose tissue-derived mesenchymal stromal cells.

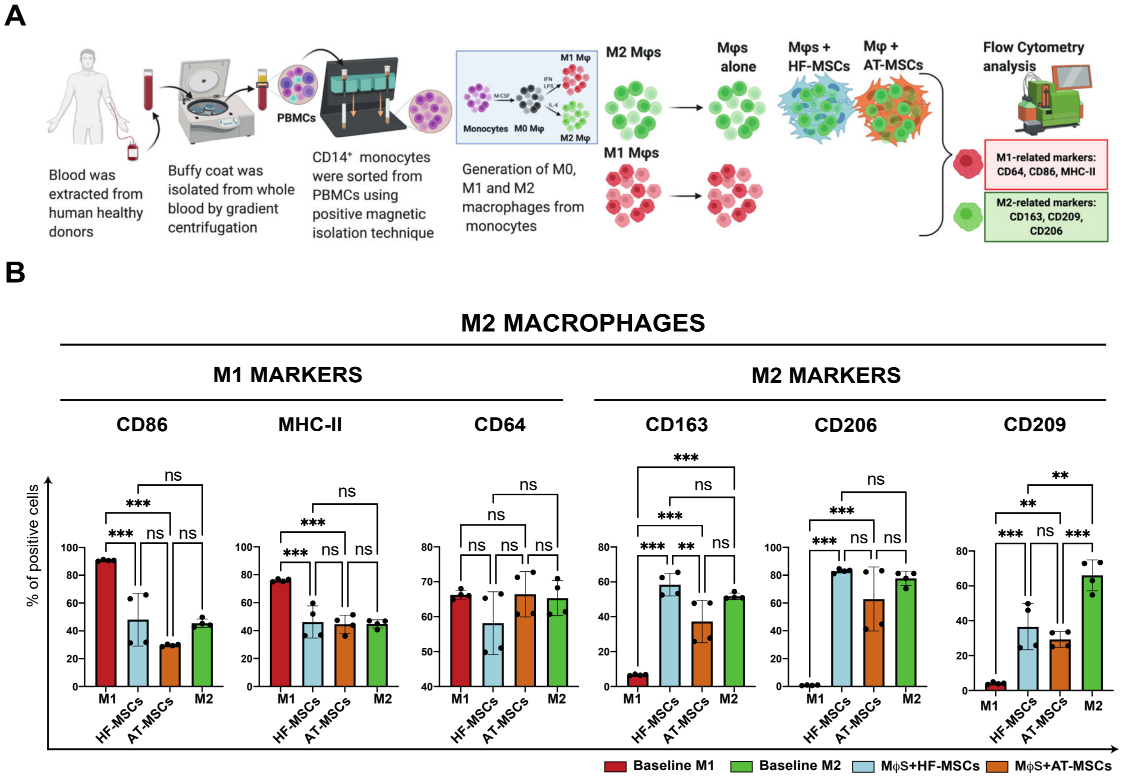

Figure S7: Effect of HF-MSCs and AT-MSCs on the modulation of M2 macrophage phenotype. (A) Human CD14$^+$ monocytes, were cultured with M-CSF for 8 days. Obtained M0 macrophages were kept in M1 or M2 differentiation conditions for 48 hours. Once all the different types of macrophages were ready, macrophages were co-culture with HF-MSC or AT-MSC for another 48 hours. (B) Bar-graphs showing M1 related phenotypic marker (CD64, CD86 and MHC-II) and M2 related phenotypic marker (CD163, CD209 and CD206) expression when M2 macrophages were co-cultured with MSCs. Each data represents the mean for at least three wells ± SD. Statistical significance: ***p < 0.001 and ** p < 0.01 when compared with macrophages without MSCs; ns: no significant differences, p > 0.05. Mϕs macrophages. PBMCs, peripheral blood mononuclear cells. HF-MSCs, hair follicle-derived mesenchymal stromal cells. AT-MSCs, adipose tissue-derived mesenchymal stromal cells.

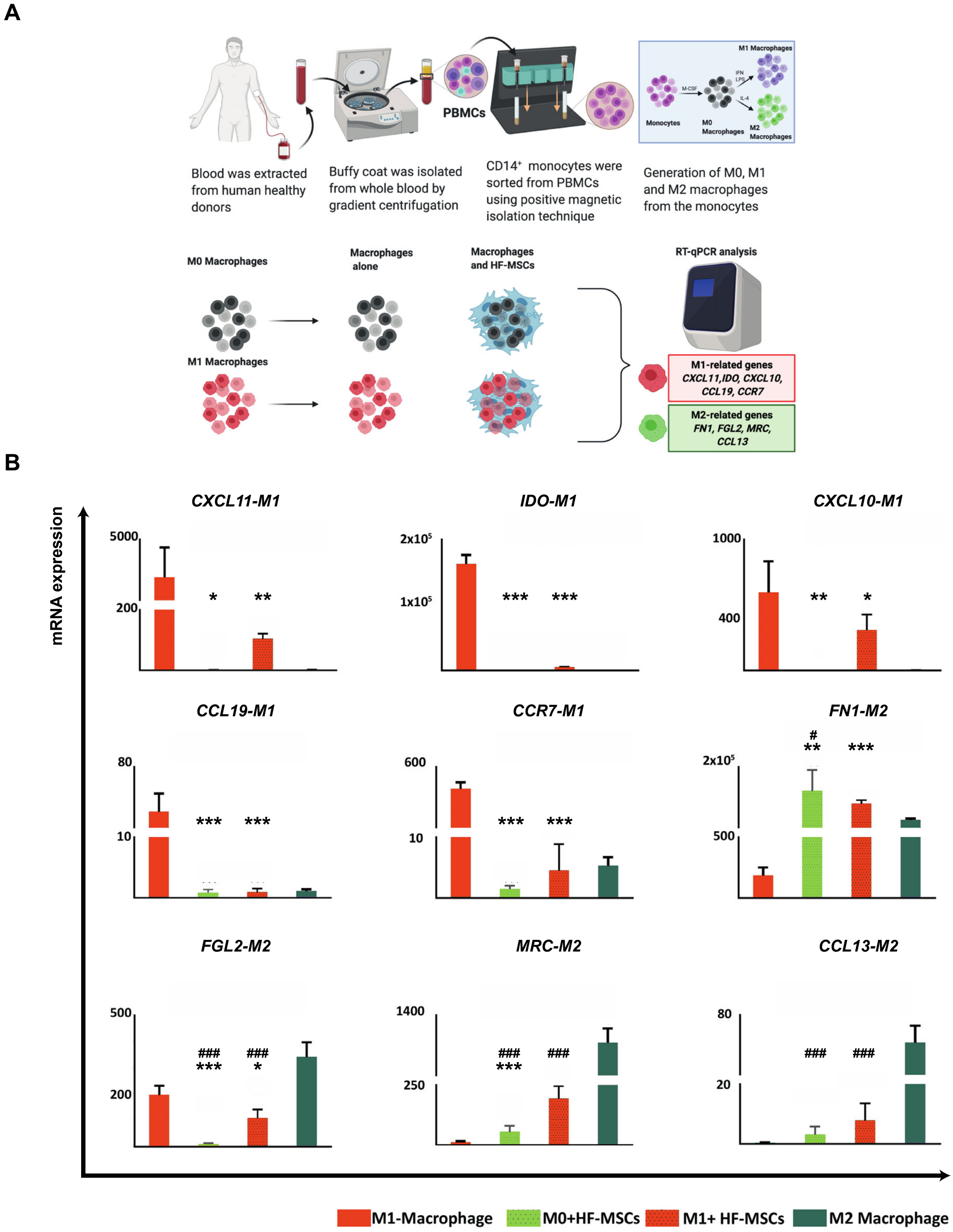

Figure S8: Effect of HF-MSCs on the gene expression of M0 and M1 macrophages. (A) CD14+ monocytes, obtained from blood samples of healthy donors were cultured with M-CSF for 8 days. Obtained M0 macrophages were kept in M1 or M2 differentiation conditions for 48 hours. Once all the different types of macrophages were ready, it was followed by the addition of HF-MSC to the culture of M0 and M1 macrophages in 1:1 ratio for another 48 hours. (B) Bar-graphs showing either M1 related gene (CXCL11, IDO, CXCL10, CCL19 and CCR7) or M2 related gene (FN-1, FGL2, MRC and CCL13) expression when M0 or M1 macrophages were cocultured with

HF-MSCs. Each data represents the mean for at least three wells ± SD. Statistical significance: ***$p < 0.001$, ** $p < 0.01$ * $p < 0.05$ when compared with M1 control. ###$p < 0.001$, #$p < 0.05$ when compared with M2 control. PBMCs, peripheral blood mononuclear cells. HF-MSCs, hair follicle-derived mesenchymal stromal cells. AT-MSCs, adipose tissue-derived mesenchymal stromal cells.
